# Congenital Paraesophageal Hernia with Intrathoracic Gastric Volvolus in Two Sisters

**DOI:** 10.5402/2011/856568

**Published:** 2011-04-19

**Authors:** Ahmed H. Al-Salem

**Affiliations:** Department of Pediatric Surgery, Maternity and Children Hospital, P.O. Box 61015, Qatif, Dammam 31911, Saudi Arabia

## Abstract

Congenital paraesophageal hernia is rare in infants and children. This paper describes our experience with seven infants and children with congenital paraesophageal hernia with emphasis on two sisters who presented with unusually large paraesophageal hernias and herniation of most of the stomach resulting in intrathoracic gastric volvolus. The literature on the subject is also reviewed.

## 1. Introduction

Paraesophageal hernias are uncommon in infants and children, and most of the reported cases are acquired resulting as a complication following Nissen's fundoplication for gastroesophageal reflux [1]. Acquired paraesophageal hernia is commonly seen in infants under the age of one year, neurologically impaired children, and in those where repair of the crura was not done at the time of fundoplication. Congenital paraesophageal hernia on the other hand is relatively rare in the pediatric age group, and most of the cases occur sporadically, but there are reports of familial paraesophageal hernias, including 3 pairs of two affected siblings [2–6]. This paper describes the 4th pair of siblings with congenital paraesophageal hernia complicated by intrathoracic gastric volvolus.

## 2. Patients and Methods

The medical records of all patients admitted to our hospital with the diagnosis of congenital paraesophageal hernia were retrospectively reviewed and the following information was obtained: age at diagnosis, sex, presenting symptoms, method of diagnosis, treatment, and outcome.

## 3. Results

Between 1990 and 2007, seven infants and children with the diagnosis of congenital paraesophageal hernia were treated at our hospital. There were 3 males and 4 females. Their age ranged from 2 days to 2.5 years (mean 16.3 months). One of our patients presented acutely, immediately after birth with respiratory distress secondary to a large congenital paraesophageal hernia. Three presented with recurrent chest infection and failure to thrive, while the remaining three had repeated attacks of vomiting with fullness and pain in the epigastrium in one of them. Another patient was a case of esophageal atresia and tracheoesophageal fistula who had repair when he was two days old. He presented at the age of 2.5 years with recurrent attacks of cough and vomiting of one-year duration. He was found to have a large left paraesophageal hernia on barium meal. He underwent upper gastrointestinal endoscopy which showed paraesophageal hernia and mild esophagitis with no stricture or stenosis. Their clinical features are shown in Table 1. Two of our patients were sisters, and both of them presented with recurrent chest infection and failure to thrive. At the time of presentation, both of them had large congenital paraesophageal hernia with intrathoracic gastric volvolus as shown below.

## 4. Case Reports

**Case 1**

**Figure 1:**
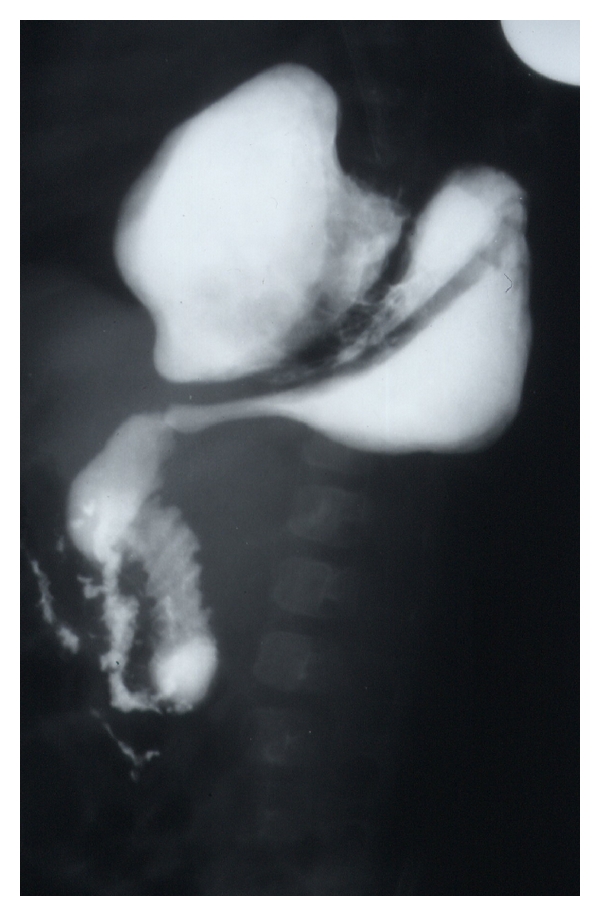
Barium meal showing a large paraesophageal hernia with herniation of the stomach into the chest and intrathoracic gastric volvolus.

A 3-month-old female was referred to our hospital with a history of repeated attacks of vomiting of one-week duration. Prior to this, she was complaining of recurrent chest infection and failure to thrive. Two days prior to presentation, the vomiting was coffee ground. Her chest X-ray and barium meal showed a large paraesophageal hernia with an intrathoracic gastric volvolus (Figure 1). She underwent an emergency surgery and was found to have a large right paraesophageal hernia with a sac containing almost the whole of the stomach. The contents were reduced, and the stomach was found congested but viable. The defect was repaired, and anterior to the abdominal wall as well as fundal to the diaphragm gastropexy were also added. Postoperatively, she developed dysphagia, and a repeat barium swallow showed narrowing at the lower esophagus most likely due to a tight hiatus. She was reoperated on and the esophageal hiatus was found to be narrow. The upper most of the sutures used to narrow the hiatus was removed. Subsequently, she did well and was discharged home in a good general condition. On followup, she was well and tolerating both solids and liquids.

**Case 2**

**Figure 2:**
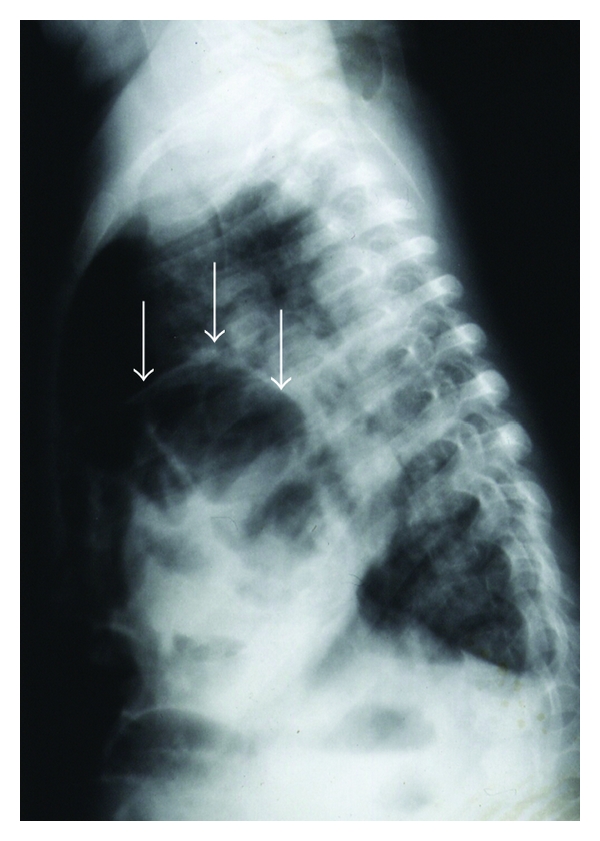
Lateral chest X-ray showing bowel herniation into the chest.

**Figure 3:**
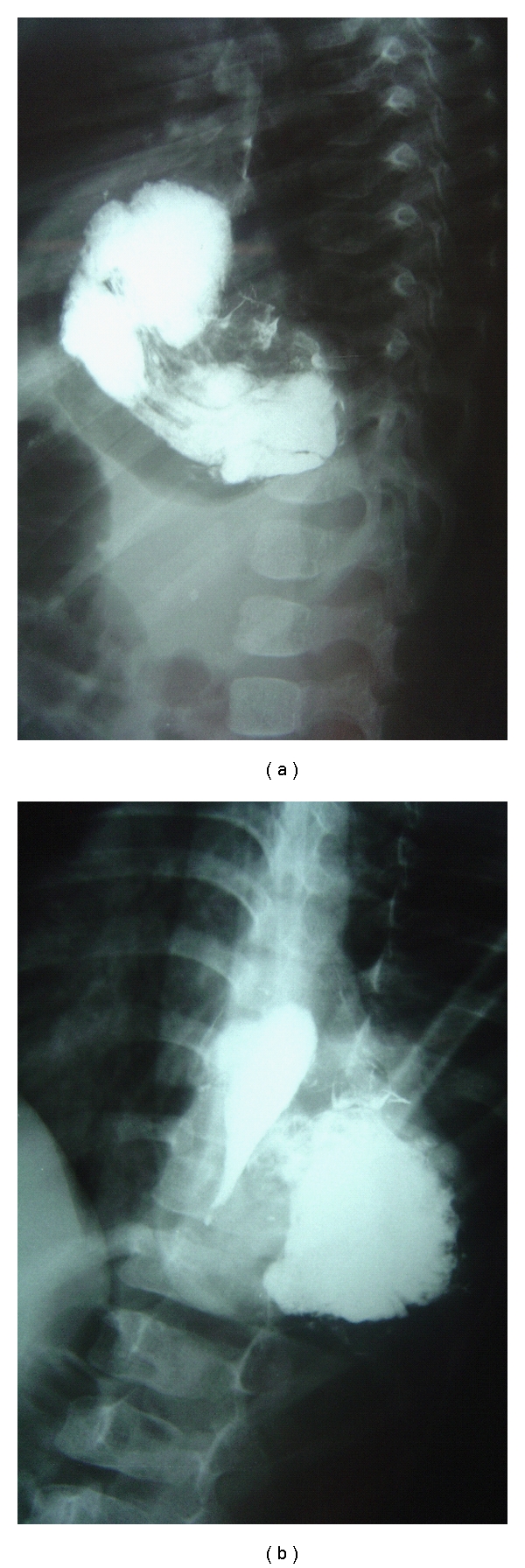
Barium meal showing herniation of the stomach into the chest with intrathoracic volvolus.

**Figure 4:**
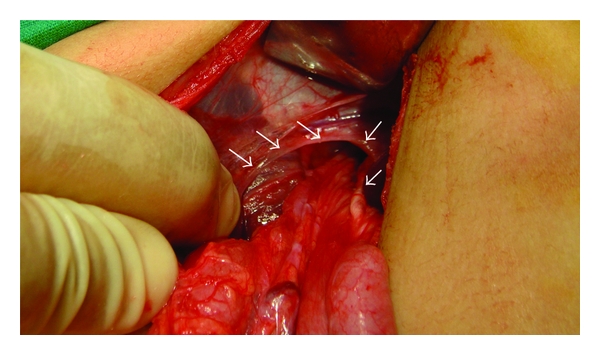
Intraoperative photograph showing a large hiatal opening after reduction of the hernia content.

1.5-year-old female was admitted to the hospital with chest infection and failure to thrive. Her chest X-ray showed herniation of bowel loops into the chest (Figure 2). Her barium meal showed herniation of most of the stomach into the chest with intrathoracic gastric volvolus (Figure 3). She was operated on and found to have a large right paraesophageal hernia with herniation of most of the stomach as well as part of the transverse colon, small bowel loops, and part of the left lobe of the liver (Figure 4). The contents were reduced, the defect was repaired, and Nissen's fundoplication was also added. Postoperatively, she did well and was discharged home in a good general condition. One week later, she was readmitted to the hospital with dysphagia, and barium swallow showed narrowing in the lower third of the esophagus. She underwent balloon dilatation of the narrowed segment and postoperatively, she did well and was discharged home in a good general condition. On followup, she was well and tolerating both liquids and solids.

## 5. Discussion

Gastric volvulus is an abnormal rotation of the stomach leading to partial or total obstruction, and according to the axis of rotation, it is classified into organoaxial, mesentericoaxial, and mixed [7–11]. In mesentericoaxial volvolus, the stomach rotates around an imaginary axis passing through the greater and lesser curvatures, while in organoaxial, the stomach rotates around an imaginary axis passing between the esophagogastric junction and the pylorus [7]. Organo-axial volvolus is the commonest. The mixed variety is extremely rare and difficult to diagnose both radiologically and intraoperatively. Gastric volvulus is rare in the pediatric age group, and depending on its presentation, it is also classified as acute where urgent surgical treatment is mandatory and chronic. Based on its location, gastric volvolus is classified into intra-abdominal and intrathoracic [7, 10]. Intrathoracic gastric volvolus is extremely rare and seen in children with diaphragmatic hernia, and intrathoracic herniation of the stomach. This was the case in both of our patients where there was a large paraesophageal hernia with herniation of the stomach and intrathoracic gastric volvolus. Gastric volvolus is also classified according to the etiology into idiopathic where no precipitating cause could be found and secondary to other anatomical defects such as diaphragmatic hernia, eventration of diaphragm, Morgagni's hernia, paraesophageal hernia and congenital asplenia [7]. 

Hiatal hernias are classified into two types: a sliding hiatal hernia and a paraesophageal hernia. Paraesophageal hernias constitute about 3.5–5% of all hiatal hernias with a female preponderance (M : F 1 : 4) [2]. Paraesophageal hernias in the pediatric age group are divided into congenital and acquired and irrespective of the type whether congenital or acquired they are relatively rare. The vast majority of paraesophageal hernias are, however, acquired commonly seen following Nissen's fundoplication for the treatment of gastroesophageal reflux [1]. By definition, paraesophageal hernia occurs when the stomach protrudes laterally through the esophageal hiatus toward the chest while the gastroesophageal junction remains in its normal anatomic position. This, however, is not the case in the pediatric age group where in most of cases the whole stomach herniates into the thoracic cavity with the gastroesophageal junction lying in the chest [4, 6, 12]. These may represent a combined type of sliding and paraesophageal hernias, or in the pediatric age group, congenital paraesophageal hernia is distinct and different from its adult counterpart. Another distinguishing feature of pediatric paraesophageal hernia is that it is not uncommon for other intra-abdominal organs to herniate into the chest through the hiatal opening. Congenital paraesophageal hernia must also be differentiated from purely intrathoracic stomach, an entity that is known to be associated with a short esophagus [13–15]. In all our patients and once the content of the hernia sac reduced, they were found to have a normal esophagus. One of the largest series of paraesophageal hernia in children was that reported by Karpelowsky et al. from the Red Cross War Memorial Children's Hospital in South Africa [4]. They reported 59 children treated over a 42-year period. These children usually present with recurrent chest infection or vague gastrointestinal symptoms. Awareness of this is important as congenital paraesophageal hernias are known to be associated with potentially lethal complications like gastric volvolus with partial or complete gastric obstruction, strangulation, and perforation [16, 17]. Two of our patients presented with paraesophageal hernia and intrathoracic gastric volvolus. It is also of importance to note that large congenital paraesophageal hernia can present at or soon after birth with respiratory distress that can be confused with the more common congenital posterolateral diaphragmatic hernia. One of our patients presented immediately after birth with respiratory distress and was found to have a large paraesophageal hernia.

The exact etiology of congenital paraesophageal hernia is not known. It is postulated that congenital paraesophageal hernia is secondary to embryonal developmental defects in the lumbar component of the diaphragm leading to defective right crus of the diaphragm [2]. A familial occurrence of hiatal hernia was first suggested in 1939 [18]. Since then there have been several reports documenting the occurrence of hiatal hernia among siblings, and an autosomal dominant mode of inheritance was suggested [19]. This was not the case for congenital paraesophageal hernia where most of the reported cases occur sporadically. There is, however, a very limited number of reports describing familial paraesophageal hernias including three pairs of affected siblings [2]. Our two siblings represent the fourth pair to be reported with familial paraesophageal hernias. This unusual familial occurrence supports a genetic predisposition to the development of congenital paraesophageal hernia. One of our patients had esophageal atresia and tracheoesophageal fistula, and this may have subsequently contributed to the development of paraesophageal hernia.

The treatment of congenital paraesophageal hernia is surgical repair. This is even for asymptomatic, incidentally discovered cases. This is to obviate the risk of gastric volvolus, strangulation, and perforation in spite of its low frequency. The addition of an antireflux procedure is still controversial. The rarity of this condition in the pediatric age group makes it difficult to evaluate the true necessity of adding an antireflux procedure for these patients. We, however, like others advocate adding an antireflux procedure at the time of hernia repair [4]. This is supported by the fact that 12 (60%) of the 20 patients in Karpelowsky et al. series who did not have fundoplication at the time of hernia repair developed recurrent reflux symptoms [4]. The recent advances in minimally invasive surgery have made it feasible and safe to repair paraesophageal hernias laparoscopically in children and adults. This is even in the presence of intrathoracic gastric volvolus [20–22].

## Figures and Tables

**Table 1:** Clinical features of paraesophageal hernia.

No.	Age	Sex	Presentation	Duration of symptoms	Associated anomalies	Site	Treatment
(1)	2 days	F	Respiratory distress	Since birth	Premature + congenital heart disease	R	Repair + Nissen's fundoplication

(2)	3 months	F	Recurrent attacks of vomiting, failure to thrive	1 week	None	R	Repair + gastropexy

(3)	1 year	F	Recurrent vomiting, epigastric pain, and fullness	3 months	Sickle cell disease	R	Repair + Nissen's fundoplication

(4)	1.5 years	F	Recurrent chest infection and failure to thrive	1 year	Congenital heart disease	R	Repair + Nissen's fundoplication

(5)	1 year and 9 months	M	Recurrent chest infection	Since 1.5 months old	None	R	Repair + Nissen's fundoplication

(6)	2.5 years	M	Recurrent chest infection	6 months	Right inguinal hernia	R	Repair

(7)	2.5 years	M	Recurrent attacks of cough and vomiting	1 year	Esophageal atresia and trachea-esophageal fistula	L	Repair + Nissen's fundoplication
